# Personalized medicine and education: the challenge

**DOI:** 10.3325/cmj.2012.53.298

**Published:** 2012-08

**Authors:** Jacques Haiech, Marie-Claude Kilhoffer

**Affiliations:** 1 School of Biotechnology, University of Strasbourg, Strasbourg, France *haiech@unistra.fr*; 2 School of Innovation, PACRI, St-Louis Institute, Paris, France; 3 School of Pharmacy, University of Strasbourg, Strasbourg, France

## Abstract

**Abstract:**

Although personalized medicine appears to be a truism, medical doctors are still generally trained in an old-fashioned manner with a focus on reactive treatment. The aim of this paper is to emphasize the evolution of life sciences into a more predictive science, where the development of quantitative models is starting to take place. Personalized medicine is a consequence of such paradigm shift. To keep up with the change, the various actors within the health system must be trained in a completely different manner, focusing on the ability to work as part of a multidisciplinary team that includes medical doctors, nurses, engineers in medical imaging, and others who collect information from patients. In addition, these teams should include modelers that are able to integrate the flood of data into predictive and quantitative models. The challenge of implementing new training methods in line with the shift is a major bottleneck to the emergence and success of personalized medicine in our societies.

Medicine has always been personal since it involves primarily the interaction between a patient and his family doctor. However, the patient was not, and still is not, necessarily at the center of the doctor's thinking. The observables are the symptoms seen by the physician or felt by the patient. The doctor’s mission is to remove or reduce them by means of adequate therapies. Faced with a symptom or set of symptoms that indicate a particular disease, the physician makes the choice of therapy without regard to the patient’s individual characteristics. In the existing paradigm, for a given disease the treatment is generally universal.

The study of the patient as a living system and the diseases that can be linked to disturbances of this system has been carried out using a reductionist approach that, based on the current status of life sciences, is still limited to a descriptive phase. Life sciences are thus a domain describing the living organisms in which the descriptive results are classified and structured but not yet used to build quantitative and predictive models (Figure 1).

**Figure 1:**
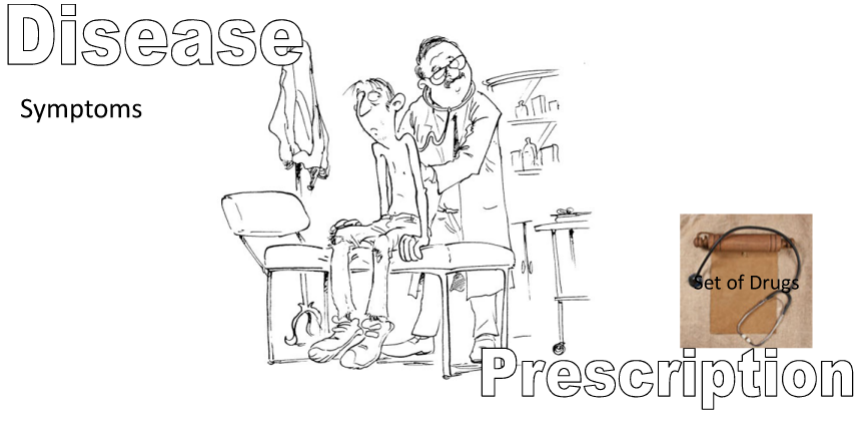
Medical doctor is linking symptoms to prescription.

This has several consequences:

1) A patient is considered to be a black box that can be observed in order to detect macroscopic observables and deviations from average behavior,

2) A patient is considered at different levels of complexity (or simplicity in reality) such as proteins, cell types, organs, organisms, without considering these elements in their globality. This leads to the impossibility of having a systemic view, and consequently the inability to either model the patient to understand the emergence of symptoms or to predict the effects of a treatment on a given patient.

3) A patient is observed for macroscopic parameters (temperature, pressure, macroscopic images, etc) or by questioning him about how he feels. This circumstance is due to the lack of technologies to observe a healthy human or a patient comprehensively and at different levels of complexity.

4) A future medical doctor is taught by observing his masters and accumulating a database of observations. He improves his medical cognition from experience.

Biology is experiencing a transition from a descriptive science to a predictive science. It is the evolution of -omics technologies that is enabling this development and accompanying its progression. In this context, biology and medicine are becoming systemic. We can, and must, consider the healthy and the sick person as a biological system that is apprehended in its totality and at all its levels of complexity.

This is a paradigm shift that leads us to consider:

1) The patient as an entity resulting from the integration of elements from the ecosystem (viral attack, xenobiotics, etc), genetic characteristics (to allow stratification of the patient population by attempting to create homogeneous groups thus facilitating stratified medicine), and individual elements (for customizing the approach to treatment which results in truly *personalized medicine*)

2) A physician to be a member of a multidisciplinary group of health care stakeholders with the aim of modeling the act of care to make it predictive for a particular patient (Figure 2).

**Figure 2:**
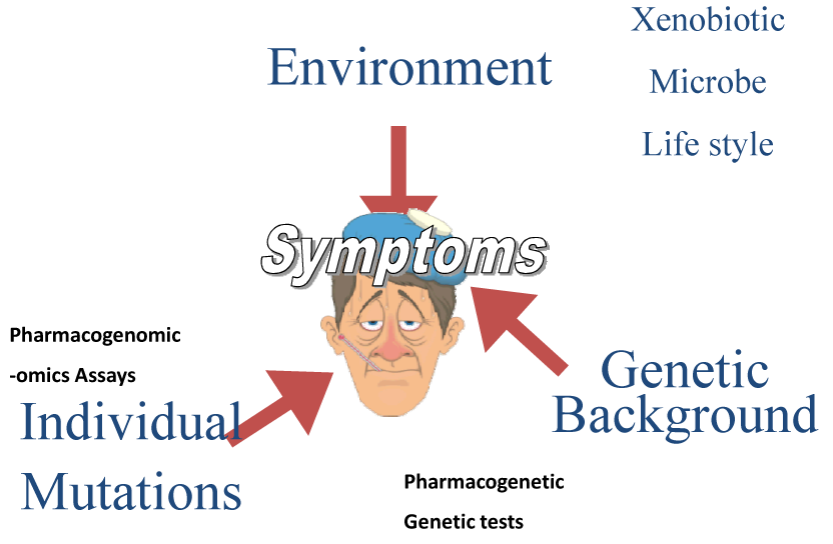
The patient is at the center of the health system.

This modeling, which should be deliberately very simple, will be composed of at least three steps that are described below in the context of a generic biological system:

1) A collecting data step where these data are heterogeneous, of variable quality and compose the patient's clinical record that in the best scenario should be computerized.

2) A second step where the interactions between system elements are described, compared to, and complemented by, existing data in public databanks or references. One goal is to have a comprehensive view of existing or predicted interactions and to then analyze the topology of the resulting interaction graph.

3) The third step is to quantify the flow of matter, energy or information between the nodes of this (these) interaction network(s).

These three steps can approach a partial or complete patient model that can be used to make rational therapeutic decisions assisted by computer technologies (or, for the health industry, to address computer assisted rational design of new therapies) (1) (Figure 3).

**Figure 3:**
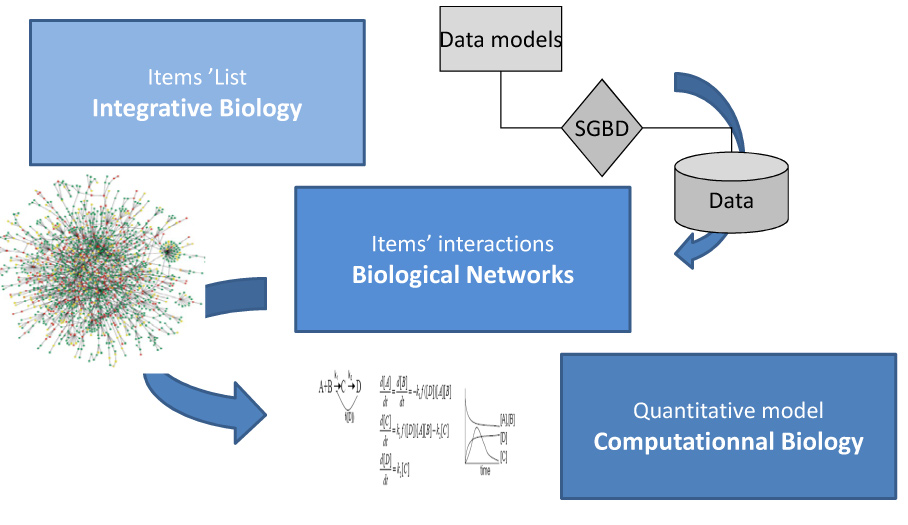
The three steps to build a qualitative and predictive model.

Training of physicians who enter the era of personalized medicine is of utmost importance and the traditional academic setting has to be adapted in the light of the new paradigm. Training courses have to be rethought in a new pedagogical space and include at least:

1) A pedestal of cognition that, in addition to medical knowledge, takes into account training in database building, graph theory, and signal theory,

2) Development of skills to allow physicians to work as a fully integrated member of a team as well as manage a project,

3) Immersion of groups of students into real projects in order to develop creativity and innovation (Figure 4).

**Figure 4:**
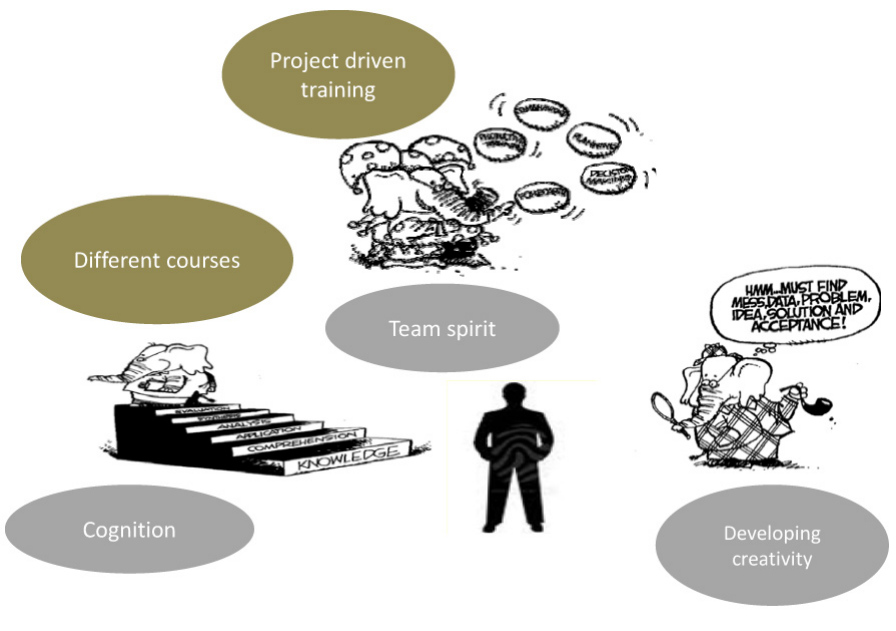
The pedagogical space.

Ideally, the curriculum of a medical doctor involved in personalized medicine, will be also customized for each student. That will necessitate a modification of the professor’s skills. The teacher must not only be a lecturer but also a coach. Indeed, a specific individual must develop specific skills to integrate with a multidisciplinary team. Therefore, although somewhat counterintuitive, acquiring a team spirit for a physician in training will require a personalized curriculum.

Another challenge for the community of professors is to encourage creativity and innovation, namely to find new methods to train modelers of biology and to induce an appreciation and appetite for modeling on the part of biologists. Some pilot experiments have been reported in a recent article in *Science* (2).

The use of serious games may also be a necessity to facilitate the building of a common language between the variety of disciplines that need to interact for the sake of personalized medicine (3,4).

In conclusion, three main challenges can be envisioned:

1) In research: when personalized medicine approaches require drugs that act on the cellular interaction network of a given individual, and not on a specific component of this network, we must be able to build models that allow us to predict the action of the drug on a specific patient or a group of patients,

2) With the society and the patient associations: the targeted therapies and the therapeutic innovations have to be discussed in order to anticipate the necessary regulations for a safe usage of those innovative therapies, and for their access to the whole population,

3) In education: the training of medical doctors but also the appreciation of new actors in the health organization that together need to change the training principles that have been applied since the time of the Ancient Greeks and the Egyptians.
